# Impact of Oil Frying on the Physicochemical Properties and Nutritional Quality of Hilsa Fish (*Tenualosa ilisha*)

**DOI:** 10.1002/fsn3.71822

**Published:** 2026-04-27

**Authors:** Md. Suman Rana, Arafat Hossain, Rafiqul Islam, Md. Pallob Hossain, Bhuiyan Mohammad Khaled, Md. Momin Khan, Sharmin Akther, Shafi Ahmed, Mrityunjoy Biswas

**Affiliations:** ^1^ Department of Food Engineering Jashore University of Science and Technology Jashore Bangladesh; ^2^ Department of Food Engineering Gopalganj Science and Technology University Gopalganj Bangladesh

**Keywords:** amino acid, fatty acid, lipid oxidation, Maillard reaction, oil frying

## Abstract

This study investigated the effects of frying duration on the physicochemical properties, nutritional composition, and sensory quality of Hilsa ( *Tenualosa ilisha* ) fried in mustard oil at 170°C–180°C for 0–12 min. Frying resulted in significant moisture loss (68.50%–41.35%) accompanied by increased fat content (12.32%–27.25%) due to oil absorption. Although protein content increased on a wet basis (18.52%–21.26%), dry‐basis protein declined markedly (58.79%–35.55%), indicating thermal degradation. Prolonged frying caused substantial reductions in essential amino acids, particularly lysine and histidine, and significant losses of polyunsaturated fatty acids including a 59% decrease in eicosapentaenoic acid (C20:5n‐3, EPA). Conversely, saturated fatty acids increased from 42.64% to 49.17%, along with a rise in trans fatty acids. Lipid oxidation intensified with frying time, as evidenced by increases in peroxide value (5.11–6.84 meq O_2_/kg) and acid value (3.95–6.85 mg KOH/g), alongside a decline in iodine value (57.34–38.68 g I_2_/100 g). Color analysis showed significant darkening (L* decreased from 65.22 to 41.24) and increased redness (a* from 2.56 to 5.32), whereas textural properties indicated increased hardness (12.50–36.52 N) and chewiness, with reduced springiness and cohesiveness. Mineral contents increased due to moisture loss and concentration effects. Sensory evaluation identified 3–6 min as the optimal frying duration, yielding the highest scores for flavor, texture, and overall acceptability.

## Graphical abstract

Frying Hilsa fish ( *Tenualosa ilisha* ) in mustard oil at 170°C–180°C for 3–6 min optimally balances sensory quality and nutritional retention. Prolonged frying progressively reduces moisture (68.5% → 41.4%), increases fat (12.3% → 27.3%), degrades EPA by 59%, and elevates lipid oxidation. Sensory analysis identified 3–6 min frying as optimal, balancing flavor, texture, and nutritional quality.

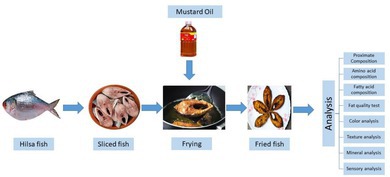

## 1 Introduction

Hilsa ( *Tenualosa ilisha* (Hamilton, 1822)) is the national fish of Bangladesh and plays a vital role in the dietary habits, culture, and fisheries of the Ganga–Brahmaputra–Padma river system (Alam et al. 2025). Its distinctive flavor, texture, and nutritional value have contributed to increasing global demand in recent years. Bangladesh is the leading producer, accounting for approximately 50%–60% of global supply, followed by Myanmar (20%–25%) and India (15%–20%) (Afsana et al. 2024; Begum et al. 2016). Due to its high market value and cultural importance, Hilsa plays a significant role in food security, livelihoods, and culinary traditions across South Asia.

Deep fat frying is one of the most widely used cooking methods worldwide because it produces foods with desirable sensory characteristics such as crispiness, appealing color, and characteristic flavor. During frying (typically 160°C–190°C), food undergoes complex heat and mass transfer processes, including rapid moisture evaporation and oil absorption, leading to structural and compositional changes such as protein denaturation, starch gelatinization, and Maillard reactions (Saguy and Dana 2003; Gertz 2014). These transformations induce physicochemical changes that can influence nutritional and sensory value.

A major limitation of frying is the combined effect of lipid oxidation and nutrient degradation. Elevated temperatures promote oxidation of lipids, particularly polyunsaturated fatty acids, resulting in the formation of hydroperoxides, aldehydes, and trans fatty acids (Dobarganes and Márquez‐Ruiz 2015). Simultaneously, essential nutrients such as amino acids and long‐chain n‐3 fatty acids are highly susceptible to thermal degradation, reducing their bioavailability and nutritional value (Goswami and Manna 2020). These processes occur concurrently and are interrelated, as oxidative reactions contribute to both lipid deterioration and nutrient loss, ultimately affecting the overall quality and shelf stability of fried foods. Moreover, frequent consumption of fried foods rich in oxidized lipids and trans fats has been associated with increased risks of obesity, cardiovascular diseases, and type‐2 diabetes (Oke et al. 2018).

The extent of these changes depends largely on frying conditions including temperature, duration, and oil composition. During frying, edible oils undergo autoxidation and hydrolysis, processes influenced by fatty acid composition and thermal exposure (Dobarganes and Márquez‐Ruiz 2015; Zhang et al. 2020). Higher temperatures and prolonged frying enhance moisture loss and promote oil uptake through water–oil exchange, thereby altering the composition and lipid content of the food (Tavares et al. 2018; Delgado‐Andrade et al. 2010; Fofandi et al. 2020). At the same time, thermal stress accelerates protein denaturation and degradation of essential amino acids, further contributing to reduced nutritional quality (Goswami and Manna 2020). These interrelated phenomena highlight the importance of controlling frying conditions to maintain both physicochemical and nutritional attributes.

Recent studies have demonstrated that nutrient retention and lipid stability during frying are governed by interactions among processing conditions, food structure, and oil characteristics (Shaziya et al. 2022; Khaled et al. 2024; Valle et al. 2024). Alternative frying techniques, such as vacuum frying and two‐stage frying, have been developed to reduce oil uptake and limit oxidative degradation. Vacuum frying, in particular, operates under reduced pressure and lower temperatures, thereby preserving both sensory attributes and nutritional quality (Juvvi et al. 2024). However, conventional deep frying remains the most commonly used method in both household and commercial settings due to its simplicity and effectiveness (Arslan et al. 2018).

The stability of frying oil is another critical factor influencing the quality of fried foods. High‐temperature frying induces oxidation, hydrolysis, and thermal degradation reactions in edible oils, leading to increased peroxide values and free fatty acids while reducing oxidative stability (Giuffrè et al. 2017, 2018). Similar effects have been observed in sunflower oil, where prolonged heating results in increased acidity and degradation of unsaturated fatty acids (Santos et al. 2017). These changes not only affect oil quality but also influence the nutritional and sensory properties of the fried product.

Mustard oil ( *Brassica juncea* L.) is widely used as a frying medium in South Asian countries including Bangladesh, India, and Nepal. It is characterized by its distinctive flavor and high content of unsaturated fatty acids, including oleic, linoleic, and α‐linolenic acids, along with bioactive compounds such as tocopherols that exhibit antioxidant properties (Chhajed et al. 2021; Khaled et al. 2024; Gunstone 2011). However, its oxidative stability during frying depends on its fatty acid composition, with polyunsaturated fatty acids being more prone to thermal degradation.

Hilsa fish is a rich source of high‐quality protein, essential amino acids, minerals, and long‐chain n‐3 polyunsaturated fatty acids such as docosahexaenoic acid (C22:6n‐3, DHA) and eicosapentaenoic acid (C20:5n‐3, EPA), which are associated with cardiovascular and metabolic health benefits (Njoroge et al. 2012; Roos et al. 2003; Mohanty et al. 2012). However, these nutrients are sensitive to heat, and their degradation during frying may reduce the overall nutritional value of the fish. Despite the widespread consumption of fried Hilsa, limited information is available on how frying duration affects its physicochemical and nutritional properties when fried in mustard oil. Therefore, this study aimed to evaluate the effects of different frying durations on the proximate composition, fatty acid profile, amino acid composition, mineral content, and sensory attributes of Hilsa fish.

## 2 Materials and Methods

### 2.1 Sample Collection and Preparation

Fresh Hilsa fish were randomly collected from the local fish market in Barishal, Bangladesh in June 2024. A total of six fish with an average weight of approximately 900–1000 g were used in the experiment. Fish of similar size and freshness were selected to minimize biological variability. The samples were transported to the Food Engineering Laboratory at Jashore University of Science and Technology in insulated ice boxes to preserve freshness prior to processing.

In the laboratory, the fish were washed with tap water, descaled, and cut into uniform pieces of approximately 3 cm thickness, with an average weight of 80–100 g per piece. Prior to frying, the initial moisture content of the raw Hilsa fish was determined to be 68.50% (wet basis) as presented in Table 1. To ensure that the results reflected only the effect of frying in mustard oil, no flour, batter, salt, or other coating ingredients were applied to the fish pieces before frying. The fish samples were fried directly without any pre‐treatment so that changes in moisture content, oil absorption, and nutrient composition could be attributed solely to the frying process.

**TABLE 1:** Proximate composition of Hilsa fish.

Parameter	Basis	Frying time				
		0 min	3 min	6 min	9 min	12 min
Moisture	Wet basis (%)	68.50 ± 0.80^a^	57.50 ± 0.80^b^	50.15 ± 0.75^c^	44.50 ± 0.65^d^	41.35 ± 0.60^e^
	Dry basis (g/100 g DM)	217.46 ± 2.32^a^	135.29 ± 1.76^b^	100.02 ± 1.45^c^	80.18 ± 1.16^d^	70.50 ± 1.03^e^
Protein	Wet basis (%)	18.52 ± 0.40^e^	19.33 ± 0.38^d^	20.12 ± 0.35^c^	20.81 ± 0.35^b^	21.26 ± 0.35^a^
	Dry basis (g/100 g DM)	58.79 ± 0.49^a^	46.09 ± 0.47^b^	40.02 ± 0.44^c^	37.24 ± 0.44^d^	35.55 ± 0.44^e^
Fat	Wet basis (%)	12.32 ± 0.50^e^	16.97 ± 0.55^d^	21.24 ± 0.60^c^	25.46 ± 0.65^b^	27.25 ± 0.65^a^
	Dry basis (g/100 g DM)	39.11 ± 0.58^e^	40.46 ± 0.67^d^	42.23 ± 0.77^c^	45.56 ± 0.86^b^	45.57 ± 0.87^a^
Ash	Wet basis (%)	0.71 ± 0.05^e^	1.33 ± 0.05^d^	1.54 ± 0.05^c^	1.79 ± 0.05^b^	1.95 ± 0.05^a^
	Dry basis (g/100 g DM)	2.25 ± 0.05^e^	3.17 ± 0.05^d^	3.06 ± 0.05^c^	3.20 ± 0.05^b^	3.26 ± 0.05^a^

*Note:* The mean ± standard deviation of three replicates is used to express the values. A substantial difference is shown by values alongside each row that don't have the same superscript (*p* < 0.05).

Frying was performed using a deep frying method in a stainless‐steel electric frying pan. Mustard oil (Radhuni brand) was used as the frying medium due to its traditional use in Bangladeshi cuisine. According to the product label, the oil was obtained through a mechanical pressing without chemical refining. The oil was supplied in food‐grade polyethylene terephthalate (PET) bottles with a volume of 1 L per bottle. The production year of the oil was 2024, which corresponded to the year in which the frying experiments were conducted. Approximately 1.5 L of mustard oil was used to ensure complete immersion of the fish pieces during frying. The frying temperature was maintained between 170°C and 180°C and continuously monitored using a thermometer to ensure consistent thermal conditions. Fish samples were fried for 3, 6, 9, and 12 min, whereas raw fish served as the control (0 min). After frying, the fish pieces were drained on absorbent paper for approximately 2 min to remove excess oil, cooled to room temperature (on average 38°C), and stored in airtight polyethylene containers at 4°C until further physicochemical, nutritional, and sensory analyses were conducted.

### 2.2 Proximate Composition Analysis

The proximate composition of raw and fried Hilsa ( *T. ilisha* ) samples was determined according to standard methods of the Association of Official Analytical Chemists (AOAC 2016).

Moisture content was determined using the oven‐drying method (AOAC Method 950.46). Approximately 5 g of homogenized fish sample was weighed into a pre‐dried moisture dish and dried in a hot air oven at 105°C until constant weight was achieved. The moisture percentage was calculated from the weight loss during drying.

Crude protein content was determined using the Kjeldahl method (AOAC Method 981.10). About 1 g of dried fish sample was digested with concentrated sulfuric acid in the presence of a catalyst mixture using a Kjeldahl digestion unit. The digested sample was then neutralized and distilled using a Kjeldahl distillation apparatus, and the liberated ammonia was titrated with a standard acid solution. The nitrogen content obtained was multiplied by a conversion factor of 6.25 to estimate crude protein.

Crude fat content was determined using the Soxhlet extraction method (AOAC Method 960.39). Approximately 3–5 g of dried sample was extracted with petroleum ether (boiling point 40°C–60°C) for 6 h in a Soxhlet extraction apparatus. After extraction, the solvent was evaporated and the remaining lipid fraction was weighed to determine the fat content.

Ash content was measured according to AOAC Method 920.153 by incinerating approximately 2 g of dried sample in a muffle furnace at 550°C for 6 h until a constant weight of white or light gray ash was obtained. The ash content was calculated as the percentage of inorganic residue remaining after combustion.

### 2.3 Amino Acid Analysis

A modified version of the Zhou et al. (2015) method was used to analyze the sample's amino acid content. Ten milliliters of 6 M HCl containing 1 mg per liter of phenol was used to dissolve 100 mg of the sample. After 2 min of flushing the solution with nitrogen gas, the container was sealed under nitrogen conditions. After that, the sealed tube was hydrolyzed for 22 h at 110°C in an oven. Following cooling, the mixture was filtered, and a rotary evaporator was used to concentrate the filtrate twice. The concentrated filtrate was then mixed with 1 mL of diluent. After the solution was filtered through a membrane, an amino acid analyzer (Eppendorf LC 3000, Hamburg, Germany) was used to measure amino acid composition. The conditions that were used with the amino acid analyzer: injection volume: 20 μL; flow rate: 0.2 mL/min; reactor temperature: 125°C; excitation: 440 nm; emission: 570 nm; program time: 84 min. The pre‐column and analytic columns used were PEEK‐Pre‐Column “LUFA” VL 00286998 07 and PEEK‐Separation Column “LUFA” L 00282 98 07, respectively. The Hydrolysate Benson Calibration Standard H, manufactured by Eppendorf‐Biotronik in Hamburg, Germany, was used for amino acid identification.

### 2.4 Fatty Acid Composition Analysis

A gas chromatography–mass spectrometry analysis was performed utilizing a Clarus 690 gas chromatograph (PerkinElmer, CA, USA) coupled with a Clarus SQ 8 C mass spectrophotometer following the methods as described by Zilani et al. (2016). The analytical column used was an Elite‐35 with a 30 m length, 0.25 mm diameter, and 0.25 μm film thickness. Using splitless mode, a pure 1 μL sample was injected. A steady flow rate of 1 mL/min of helium (99.99%) was utilized for 40 min of operation. The material was examined using high energy (70 eV) electron ionization (EI) mode. Although the intake temperature remained constant at 280°C, the temperature of the column oven was set at 60°C for 0 min, then increased to 240°C at a rate of 5°C per minute and held for 4 min. By matching the sample chemicals to the NIST database, their identities were determined.

### 2.5 Fat Quality Analysis

The saponification value was determined following the method by Machewad et al. (2021). The extracted oil from fried fish was filtered to remove any impurities or moisture. One gram of oil was mixed with alcoholic KOH and a few glass beads in a conical flask fitted with an air condenser. The mixture was heated for 1 h, then cooled. The flask was rinsed with neutral ethyl alcohol, and the solution was titrated with HCl using phenolphthalein as an indicator. A blank (without oil) was also tested.

Acid value was analyzed as per the method followed by Khaled et al. (2024). About 2.5 g of extracted oil from fish was mixed with hot 5% ethanol and boiled. A few drops of phenolphthalein indicator were added, and the mixture was titrated with 0.1 M KOH until a light pink color stayed for a few seconds.

The iodine value was measured using a modified method by Kayanan and Sagum (2021). About 0.6 g of extracted oil was mixed with carbon tetrachloride and Wij's solution and kept in the dark for 30 min. Potassium iodide and water were then added, and the solution was titrated with 0.1 N sodium thiosulfate using starch as an indicator.

The peroxide value was determined using the method by Naik et al. (2020). One gram of extracted oil was mixed with potassium iodide, glacial acetic acid, and chloroform, then heated. After adding more potassium iodide and water, the solution was titrated with 0.002 M sodium thiosulfate using starch as an indicator. Blank titrations were done for accuracy.

### 2.6 Color Analysis

The color of raw and fried Hilsa fish was evaluated using a Precision Colorimeter (BCM‐110, China), calibrated with standard black and white ceramic tiles. Color was quantified in three dimensions: L*, a*, and b*. The L* value reflects lightness, with higher values indicating lighter samples. The a* value represents the red–green spectrum, where negative values indicate green and positive values indicate red. The b* value corresponds to the yellow–blue spectrum, with positive values indicating yellow and negative values indicating blue, allowing precise and objective color assessment of the samples.

### 2.7 Mineral Analysis

The mineral content (calcium, zinc, iron, phosphorus, magnesium, sodium, and potassium) of raw and fried Hilsa fish was determined using Inductively Coupled Plasma‐Optical Emission Spectrometry (ICP‐OES) following Islam et al. (2020). Samples were first ashed at 550°C to remove organic matter. The resulting ash was digested with a 4:1 mixture of HNO_3_ and H_2_O_2_ at 200°C until a clear solution formed. Digests were cooled, filtered through Whatman No. 1 paper, and diluted to 50 mL with deionized water. Solutions were stored at 4°C until analysis. Mineral concentrations were reported as mg/100 g wet weight.

### 2.8 Texture Analysis

The texture profile analysis (TPA) of raw and fried Hilsa samples was conducted using a TA‐XT2i texture analyzer (Stable Micro Systems, UK) fitted with a 50 N load cell and a cylindrical probe (10 mm diameter) to simulate mastication by teeth as described by Yu et al. (2020). The raw and fried Hilsa fish cubes measuring 20 × 20 × 20 mm were placed on the platform and compressed to a depth of 14 mm at a speed of 60 mm·min^−1^, with a trigger force of 0.3 N. Textural parameters, including hardness, cohesiveness, springiness, and chewiness, were recorded for three replicates.

### 2.9 Sensory Evaluation

Fish samples underwent a sensory analysis to evaluate their color, flavor, texture, taste, and overall appeal by a semi‐trained panel of 25 tasters aged between 19 and 35 years. The panel team was formed consisting of academic staff and students of the Department of Food Engineering, Jashore University of Science and Technology. The members were experts with different types of foods and capable of detecting the deviation of taste effectively. The evaluation utilized a 9‐point hedonic scale (Hooda and Jood 2005). Panelists graded the samples as follows: 9 for extremely favorable, 8 for very good, 7 for good, 6 for somewhat favorable, 5 for neutral, 4 for somewhat unfavorable, 3 for fairly unfavorable, 2 for strongly unfavorable, and 1 for extremely unfavorable.

### 2.10 Statistical Analysis

Each experiment was conducted in a triplicate manner to check quality and consistency, and the results were represented as mean ± standard deviation. IBM SPSS version 21 (SPSS Inc., Chicago, IL) was employed as the statistical data analysis tool, and ANOVA procedures were followed to determine statistical significance. The significance between the means was analyzed using the Least Significant Difference (LSD) at the 5% significance level.

## 3 Results and Discussion

### 3.1 Changes in Proximate Composition of Hilsa Fish During Frying

The proximate composition of Hilsa fish showed notable changes after frying in mustard oil, with differences observed between wet and dry basis measurements (Table 1). Frying significantly influences moisture, protein, and fat content (*p* < 0.05), primarily due to heat‐induced mass transfer and compositional changes within the fish matrix (Manral et al. 2008; Tadesse et al. 2020).

The reduction in moisture content during frying was statistically significant (*p* < 0.05) and can be attributed to rapid water evaporation at elevated temperatures. This dehydration process is further enhanced by water–oil exchange, where moisture migrates from the interior of the fish to the surface and is progressively replaced by oil. As frying time increases, changes in interfacial tension and oil polarity facilitate greater oil absorption into the tissue (Ghidurus et al. 2010). Similar moisture loss patterns in fried fish have been widely reported and are associated with increasing temperature and frying duration (Tavares et al. 2018; Fofandi et al. 2020). The loss of moisture also leads to a relative concentration of other components such as protein, fat, and ash on a wet basis.

The observed variation in protein content between wet and dry basis also showed significant differences (*p* < 0.05), reflecting the combined effects of moisture loss and thermal degradation. Although protein appears to increase on a wet basis due to concentration effects, the decrease on a dry basis indicates actual protein denaturation and degradation under prolonged heat exposure. High frying temperatures can disrupt protein structure, reduce solubility, and decrease the availability of essential amino acids. Comparable findings have been reported in fried fish, where heat‐induced protein degradation results in reduced nutritional quality (Negara et al. 2021).

Fat content increased significantly (*p* < 0.05) during frying, primarily due to oil uptake, which compensates for moisture loss and contributes to the overall lipid content of the fish. This process is driven by the formation of pores and structural changes in the muscle tissue, allowing oil to penetrate and accumulate within the matrix. Although oil absorption increases lipid content, high frying temperatures may simultaneously promote the degradation of polyunsaturated fatty acids, leading to a relative increase in more stable saturated fractions. Similar trends have been reported in previous studies, where frying resulted in increased fat content alongside alterations in lipid composition (Delgado‐Andrade et al. 2010; Tavares et al. 2018; Fofandi et al. 2020; Marimuthu et al. 2011).

Ash content also increased significantly (*p* < 0.05), mainly due to the concentration effect resulting from moisture loss rather than an actual increase in mineral content. Interactions between fish tissue and the frying medium may also contribute to minor variations. Similar statistically significant increases in ash content have been observed in fried fish with prolonged frying duration (Tadesse et al. 2020).

### 3.2 Changes in Amino Acid Composition of Hilsa Fish During Frying

The frying of Hilsa fish in mustard oil induced significant changes in amino acid composition (*p* < 0.05) (Table 2). Although some amino acids showed increasing trends, others declined with prolonged frying, indicating that thermal processing differentially affects protein structure and amino acid stability.

**TABLE 2:** Changes in amino acids of Hilsa fish.

Amino acid	Frying time				
	0 min	3 min	6 min	9 min	12 min
Aspartic acid	10.78 ± 0.30^e^	11.42 ± 0.27^d^	12.05 ± 0.25^c^	12.85 ± 0.23^b^	13.61 ± 0.21^a^
Glutamic acid	17.80 ± 0.50^e^	18.31 ± 0.44^d^	18.92 ± 0.41^c^	19.82 ± 0.39^b^	20.52 ± 0.37^a^
Glycine	4.25 ± 0.12^e^	5.56 ± 0.13^d^	6.12 ± 0.14^c^	6.70 ± 0.15^b^	7.23 ± 0.16^a^
Tyrosine	2.91 ± 0.08^e^	3.32 ± 0.08^d^	3.71 ± 0.09^c^	4.15 ± 0.10^b^	4.62 ± 0.11^a^
Phenylalanine	3.92 ± 0.11^e^	4.34 ± 0.11^d^	4.85 ± 0.12^c^	5.33 ± 0.13^b^	5.85 ± 0.14^a^
Arginine	2.45 ± 0.07^e^	2.72 ± 0.07^d^	3.13 ± 0.08^c^	3.45 ± 0.09^b^	3.91 ± 0.10^a^
Threonine	4.97 ± 0.14^a^	4.53 ± 0.13^b^	4.11 ± 0.11^c^	3.42 ± 0.09^d^	2.83 ± 0.08^e^
Serine	6.81 ± 0.19^a^	5.51 ± 0.16^b^	4.53 ± 0.13^c^	3.74 ± 0.11^d^	2.42 ± 0.07^e^
Alanine	7.43 ± 0.21^a^	6.55 ± 0.18^b^	5.55 ± 0.16^c^	4.72 ± 0.14^d^	3.63 ± 0.11^e^
Cysteine	1.56 ± 0.04^a^	1.45 ± 0.04^a^	1.22 ± 0.03^b^	0.93 ± 0.03^c^	0.65 ± 0.02^d^
Proline	1.79 ± 0.05^a^	1.52 ± 0.04^b^	1.25 ± 0.04^c^	1.09 ± 0.03^d^	0.75 ± 0.02^e^
Valine	4.38 ± 0.12^a^	3.71 ± 0.11^b^	3.17 ± 0.09^c^	2.35 ± 0.07^d^	1.56 ± 0.05^e^
Methionine	3.17 ± 0.09^a^	2.55 ± 0.07^b^	2.06 ± 0.06^c^	1.52 ± 0.04^d^	1.12 ± 0.03^e^
Isoleucine	3.65 ± 0.10^a^	3.22 ± 0.09^b^	2.62 ± 0.08^c^	2.11 ± 0.06^d^	1.43 ± 0.04^e^
Leucine	8.53 ± 0.24^a^	6.83 ± 0.19^b^	5.61 ± 0.16^c^	4.53 ± 0.13^d^	3.42 ± 0.10^e^
Histidine	5.74 ± 0.16^a^	4.35 ± 0.12^b^	3.32 ± 0.10^c^	2.32 ± 0.07^d^	1.32 ± 0.04^e^
Lysine	5.16 ± 0.15^a^	3.82 ± 0.11^b^	2.94 ± 0.08^c^	2.04 ± 0.06^d^	1.11 ± 0.03^e^

*Note:* The mean ± standard deviation of three replicates is used to express the values. A substantial difference is shown by values alongside each row that don't have the same superscript (*p* < 0.05).

The observed increase in certain amino acids, particularly aspartic acid, glutamic acid, and glycine, can be attributed to partial protein breakdown and the release of free amino acids during thermal denaturation. Glutamic acid, a key contributor to umami taste, may enhance the flavor profile of fried fish, whereas the increase in glycine suggests collagen degradation within connective tissues (Erkan et al. 2010). Similarly, increases in aromatic amino acids such as tyrosine and phenylalanine may result from protein unfolding and exposure of previously buried residues during heating.

In contrast, several essential and heat‐sensitive amino acids showed significant reductions (*p* < 0.05) with increasing frying time. Sulfur‐containing amino acids (cysteine and methionine) and branched‐chain amino acids (valine, leucine, and isoleucine) were particularly susceptible to thermal degradation. These reductions are likely associated with Maillard reactions and oxidative processes that generate flavor compounds at the expense of nutritional quality (Goswami and Manna 2020). The decline in threonine, serine, and lysine further reflects their involvement in heat‐induced cross‐linking and browning reactions. Notably, the substantial reduction in essential amino acids such as histidine and lysine indicates a potential decrease in protein nutritional value with prolonged frying.

The degradation of proline suggests structural breakdown of connective tissues, which may contribute to textural softening of the fish. Meanwhile, the reduction of alanine and valine may be linked to Strecker degradation, a reaction pathway that produces volatile compounds such as pyrazines and sulfides, contributing to the characteristic aroma of fried fish (Özyurt et al. 2011).

Previous studies have reported variable effects of thermal processing on fish proteins and amino acids. Although some studies observed minimal changes following boiling and frying, others reported significant reductions in protein quality due to degradation and decreased bioavailability of essential amino acids (Ismail et al. 2004; Oluwaniyi et al. 2010). The present findings are consistent with these reports, confirming that prolonged frying can lead to significant alterations in amino acid composition, with implications for both nutritional value and sensory properties.

### 3.3 Changes in Fatty Acid Composition of Hilsa Fish During Frying

The frying process significantly altered the fatty acid composition of Hilsa fish (*p* < 0.05) (Table 3). A marked reduction in polyunsaturated fatty acids (PUFAs) and a relative increase in saturated fatty acids (SFAs) and trans fatty acids were observed, indicating deterioration in lipid quality during frying. These changes are primarily associated with the thermal instability of unsaturated fatty acids at high temperatures. Long‐chain fatty acids such as α‐linolenic acid (C18:3n‐3) and eicosapentaenoic acid (C20:5n‐3, EPA) are particularly susceptible to oxidative degradation due to their multiple double bonds (Sioen et al. 2006; Larsen et al. 2010). Similar statistically significant reductions in PUFAs during frying have been reported in fish and other lipid‐rich foods (Zhang et al. 2013; Wang et al. 2015; Valle et al. 2024).

**TABLE 3:** Changes in fatty acid composition of Hilsa fish.

Fatty acid		Frying time				
		0 min	3 min	6 min	9 min	12 min
Saturated fatty acids (SFA)	C11:0	0.17 ± 0.02^b^	0.19 ± 0.02^ab^	0.21 ± 0.03^ab^	0.22 ± 0.03^ab^	0.23 ± 0.04^a^
	C13:0	1.71 ± 0.02^c^	1.92 ± 0.02^bc^	2.11 ± 0.04^b^	2.31 ± 0.06^ab^	2.55 ± 0.07^a^
	C14:0	30.22 ± 0.38^b^	30.46 ± 0.30^b^	31.53 ± 0.40^a^	31.98 ± 0.52^a^	32.17 ± 0.66^a^
	C15:0	1.47 ± 0.02^a^	1.22 ± 0.01^b^	1.13 ± 0.08^b^	0.95 ± 0.01^c^	0.82 ± 0.02^d^
	C16:0	1.22 ± 0.02^d^	1.34 ± 0.01^cd^	1.44 ± 0.02^c^	1.71 ± 0.03^b^	2.23 ± 0.05^a^
	C18:0	6.43 ± 0.08^c^	6.61 ± 0.06^bc^	7.12 ± 0.09^ab^	7.47 ± 0.12^a^	7.76 ± 0.16^a^
	C20:0	0.18 ± 0.02^a^	0.21 ± 0.02^a^	0.27 ± 0.01^a^	0.29 ± 0.05^a^	0.33 ± 0.01^a^
	C27:0	1.24 ± 0.02^c^	1.53 ± 0.02^c^	2.12 ± 0.05^b^	2.54 ± 0.07^ab^	3.08 ± 0.09^a^
	∑SFA	42.64 ± 0.6^c^	43.48 ± 0.5^c^	45.93 ± 0.71^b^	47.47 ± 0.9^ab^	49.17 ± 1.10^a^
Monounsaturated fatty acids (MUFA)	C14:1	0.47 ± 0.06^a^	0.29 ± 0.03^b^	0.22 ± 0.03^bc^	0.19 ± 0.02^bc^	0.17 ± 0.02^c^
	C16:1	6.35 ± 0.13^a^	2.75 ± 0.05^b^	2.12 ± 0.03^bc^	1.47 ± 0.02^bc^	1.43 ± 0.01^c^
	C17:1	2.06 ± 0.04^a^	1.66 ± 0.02^b^	1.45 ± 0.02^bc^	1.15 ± 0.02^c^	1.02 ± 0.01^c^
	C18:1	24.37 ± 0.30^b^	24.65 ± 0.24^b^	25.42 ± 0.32^ab^	26.24 ± 0.43^ab^	26.62 ± 0.56^a^
	C22:1n‐9	2.71 ± 0.06^a^	2.66 ± 0.04^ab^	2.43 ± 0.03^b^	2.41 ± 0.03^b^	2.31 ± 0.03^b^
	C24:1n‐9	3.47 ± 0.04^a^	3.45 ± 0.03^ab^	2.36 ± 0.03^ab^	1.47 ± 0.06^b^	1.41 ± 0.06^b^
	C26:1n‐9	0.97 ± 0.01^a^	0.88 ± 0.08^ab^	0.79 ± 0.01^ab^	0.36 ± 0.01^b^	0.14 ± 0.03^b^
	∑MUFA	40.40 ± 0.4^c^	36.34 ± 0.4^c^	34.79 ± 0.5^bc^	33.29 ± 0.61^c^	33.10 ± 0.8^a^
Polyunsaturated fatty acids (PUFA)	C18:3n‐3	8.57 ± 0.11^a^	6.23 ± 0.08^b^	5.53 ± 0.11^c^	4.41 ± 0.14^d^	3.69 ± 0.18^e^
	C20:5n‐3	1.64 ± 0.08^a^	1.56 ± 0.08^a^	1.09 ± 0.01^b^	0.86 ± 0.03^bc^	0.67 ± 0.03^c^
	∑PUFA	10.21 ± 0.28^b^	7.79 ± 0.3^b^	6.62 ± 0.3^ab^	5.27 ± 0.25^a^	4.36 ± 0.25^a^
Trans fats	C18:1 t	0.22 ± 0.03^c^	0.29 ± 0.03^c^	0.47 ± 0.06^b^	0.50 ± 0.08^b^	0.55 ± 0.09^a^

*Note:* The mean ± standard deviation of three replicates is used to express the values. A substantial difference is shown by values alongside each row that don't have the same superscript (*p* < 0.05).

The apparent increase in SFAs does not indicate their formation but rather reflects the preferential degradation of unsaturated fatty acids. Since PUFAs and monounsaturated fatty acids (MUFAs) are more prone to oxidation, their reduction results in a relative shift toward more stable saturated fractions (Dobarganes and Márquez‐Ruiz 2015; Gertz 2014). This phenomenon has been widely documented in fried foods, where statistically significant decreases in unsaturated fatty acids are accompanied by an increase in SFAs (Zhang et al. 2013).

In addition to oxidative degradation, oil absorption plays a critical role in modifying the fatty acid profile. The rapid loss of moisture during frying creates structural changes in the fish tissue, facilitating oil penetration and lipid exchange between the food and the frying medium (Saguy and Dana 2003). As a result, the final fatty acid composition is influenced not only by degradation of endogenous lipids but also by the composition of the frying oil. Previous studies have confirmed that this oil–food interaction significantly determines the lipid profile of fried products under varying frying conditions (Valle et al. 2024).

The type of frying oil further influences lipid stability due to differences in fatty acid composition. Oils rich in monounsaturated fatty acids, such as canola oil, exhibit greater oxidative stability, whereas oils with higher polyunsaturated fatty acid content, such as sunflower oil, are more susceptible to thermal degradation. Consequently, frying with PUFA‐rich oils may lead to greater losses of unsaturated fatty acids, whereas more stable oils can better preserve lipid quality (Saguy and Dana 2003; Valle et al. 2024).

A significant increase in trans fatty acids (*p* < 0.05) with extended frying time indicates the occurrence of thermal isomerization. Although present at relatively low levels, the formation of trans fats is nutritionally important due to their association with adverse health effects (Wang et al. 2015; Dobarganes and Márquez‐Ruiz 2015). Prolonged heating promotes the conversion of cis double bonds to trans configurations, thereby reducing the nutritional value of the lipid fraction.

Furthermore, frying induces complex lipid reactions including oxidation, hydrolysis, and polymerization, leading to the formation of secondary products such as aldehydes and ketones (Gertz 2014; Valle et al. 2024). These compounds not only degrade nutritional quality but also influence flavor and safety. The combined effects of oxidative degradation, oil absorption, oil composition, and thermal isomerization explain the overall changes in fatty acid composition observed in this study.

Fish is widely recognized as a valuable component of a healthy diet due to its high‐quality protein and rich content of long‐chain n‐3 polyunsaturated fatty acids such as EPA and DHA. These fatty acids are associated with cardiovascular protection, improved cognitive function, and anti‐inflammatory effects. Recent studies have further emphasized the role of fish‐derived nutrients in reducing the risk of chronic diseases (Chamorro et al. 2026; Muñoz et al. 2024). Therefore, the significant reduction in n‐3 fatty acids observed during frying is of particular concern, as it may compromise the nutritional and health benefits associated with fish consumption.

### 3.4 Changes in Fat Quality of Hilsa Fish During Frying

Table 4 presents the changes in fat quality parameters including saponification, acid, iodine, and peroxide values, of oil extracted from Hilsa fish during frying. These parameters showed significant variations (*p* < 0.05) with increasing frying time, indicating progressive lipid degradation and oxidation.

**TABLE 4:** Change of fat quality of Hilsa fish.

Frying period of Hilsa fish	Saponification value (mg KOH/g)	Acid value (mg KOH/g)	Iodine value (mg I_2_/100g)	Peroxide value (meq. O_2_/kg oil)
0 min	187.21 ± 3.67^e^	3.95 ± 0.12^e^	57.34 ± 0.09^a^	5.11 ± 0.06^e^
3 min	195.44 ± 3.67^d^	4.26 ± 0.12^d^	52.78 ± 0.09^b^	5.67 ± 0.06^d^
6 min	213.48 ± 1.52^c^	4.85 ± 0.09^c^	48.57 ± 0.05^c^	5.94 ± 0.13^c^
9 min	219.50 ± 0.55^b^	5.52 ± 0.08^b^	42.27 ± 0.10^d^	6.31 ± 0.12^b^
12 min	238.09 ± 0.34^a^	6.85 ± 0.10^a^	38.68 ± 0.11^e^	6.84 ± 0.08^a^

*Note:* The mean ± standard deviation of three replicates is used to express the values. A substantial difference is shown by values inside each column that don't have the same superscript (*p* < 0.05).

The saponification value increased significantly (*p* < 0.05) with prolonged frying, reflecting the breakdown of triglycerides into smaller fatty acid molecules. This increase suggests enhanced hydrolysis and thermal degradation of lipids under high‐temperature conditions. Similar trends have been reported in fried fish and edible oils, where prolonged heating leads to the formation of lower molecular weight fatty acids (Ghidurus et al. 2010; Manral et al. 2008; Hosseini et al. 2014).

The acid value also increased significantly (*p* < 0.05), indicating the accumulation of free fatty acids as a result of lipid hydrolysis. This rise is commonly associated with the onset of rancidity and deterioration in oil quality during frying. Previous studies have similarly reported that extended frying promotes free fatty acid formation, thereby reducing the stability and acceptability of frying oils (Tadesse et al. 2020).

In contrast, the iodine value showed a significant decrease (*p* < 0.05), indicating a reduction in the degree of unsaturation of fatty acids. This decline is attributed to oxidative degradation of unsaturated lipids at high temperatures, leading to the formation of more stable saturated compounds. Comparable reductions in iodine value during frying have been widely documented, confirming the susceptibility of unsaturated fatty acids to thermal oxidation (Gertz 2014; Delgado‐Andrade et al. 2010).

The peroxide value increased significantly (*p* < 0.05) with frying time, reflecting the formation of primary oxidation products such as hydroperoxides. This increase indicates progressive lipid peroxidation and deterioration of oil. Similar findings have been reported in previous studies, where prolonged frying enhanced oxidative degradation of lipids (Goswami and Manna 2020).

### 3.5 Color Analysis of Hilsa Fish During Frying

The color parameters of Hilsa fish, expressed as L*, a*, and b* values, showed significant changes (*p* < 0.05) with increasing frying time (Table 5), indicating progressive alterations in quality.

**TABLE 5:** Color analysis of Hilsa fish.

Color parameter	Frying time				
	0 min	3 min	6 min	9 min	12 min
L*	65.22 ± 0.56^a^	58.68 ± 0.51^b^	51.38 ± 0.48^c^	45.76 ± 0.44^d^	41.24 ± 0.35^e^
a*	2.56 ± 0.12^e^	3.23 ± 0.17^d^	4.02 ± 0.12^c^	4.74 ± 0.17^b^	5.32 ± 0.12^a^
b*	23.53 ± 0.51^a^	21.05 ± 0.48^b^	18.24 ± 0.44^c^	15.49 ± 0.35^d^	12.89 ± 0.35^e^

*Note:* The mean ± standard deviation of three replicates is used to express the values. A substantial difference is shown by values inside each row that don't have the same superscript (*p* < 0.05).

The lightness (L*) value decreased significantly (*p* < 0.05), reflecting pronounced darkening of the fish surface during frying. This reduction is primarily attributed to moisture loss and the Maillard reaction, where reducing sugars react with amino acids to form brown melanoidin pigments under high‐temperature conditions. In addition, surface dehydration and crust formation further contribute to reduced light reflectance. Similar decreases in lightness during frying have been reported in fish and other fried products (Jaeger et al. 2010; Vieira et al. 2018).

In contrast, redness (a*) values increased significantly (*p* < 0.05), indicating the development of characteristic reddish‐brown coloration. This change is associated with Maillard browning reactions and lipid oxidation, both of which generate colored compounds that intensify red hues in fried foods (Zamora and Hidalgo 2011). The formation of these pigments contributes to the desirable appearance typically associated with fried products.

The yellowness (b*) value showed a significant decline (*p* < 0.05) with prolonged frying time. This decrease may be attributed to the degradation or transformation of yellow pigments and the dominance of darker brown compounds formed during thermal processing. Similar reductions in b* values have been observed in fried fish, indicating progressive pigment degradation and browning reactions (Vieira et al. 2018).

### 3.6 Minerals Analysis of Hilsa Fish During Frying

The mineral composition of Hilsa fish showed significant changes (*p* < 0.05) during frying, with an overall increasing trend observed across all measured minerals (Table 6). These increases were consistent throughout the frying period and reflect changes in concentration rather than actual mineral enrichment.

**TABLE 6:** Mineral analysis of Hilsa fish.

Mineral (mg/100 g)	Frying time				
	0 min	3 min	6 min	9 min	12 min
Calcium	344.35 ± 2.52^e^	404.38 ± 2.64^d^	446.39 ± 2.82^c^	489.14 ± 3.06^b^	519.49 ± 3.23^a^
Phosphorus	323.48 ± 3.55^e^	372.97 ± 3.82^d^	404.84 ± 4.05^c^	432.78 ± 4.24^b^	452.24 ± 4.32^a^
Iron	5.66 ± 0.05^e^	6.13 ± 0.05^d^	6.77 ± 0.05^c^	7.22 ± 0.05^b^	7.70 ± 0.06^a^
Zinc	0.82 ± 0.03^e^	1.05 ± 0.03^d^	1.08 ± 0.03^c^	1.15 ± 0.03^b^	1.22 ± 0.03^a^
Magnesium	18.82 ± 1.06^e^	20.39 ± 1.06^d^	22.21 ± 1.12^c^	23.68 ± 1.15^b^	24.95 ± 1.25^a^
Sodium	68.09 ± 1.23^e^	77.07 ± 1.33^d^	82.54 ± 1.43^c^	88.13 ± 1.54^b^	92.46 ± 1.63^a^
Potassium	345.41 ± 5.05^e^	380.24 ± 5.27^d^	402.88 ± 5.44^c^	423.55 ± 5.62^b^	440.81 ± 5.71^a^

*Note:* The mean ± standard deviation of three replicates is used to express the values. A substantial difference is shown by values inside each row that don't have the same superscript (*p* < 0.05).

The observed rise in mineral content is primarily attributed to the substantial reduction in moisture during frying, which concentrates non‐volatile components such as minerals within the fish tissue. As water is lost through evaporation, the relative proportion of minerals increases, leading to higher measured values on a wet basis. This concentration effect is a well‐established phenomenon in thermally processed foods (Yu et al. 2020).

In addition to moisture loss, minor interactions between the fish tissue and frying medium may contribute to slight variations in mineral composition. However, the dominant factor influencing the observed increases remains dehydration rather than mineral gain. Similar statistically significant increases in mineral content during frying have been reported in previous studies, where elevated levels of calcium, phosphorus, iron, zinc, and other minerals were associated with moisture reduction (Rosa et al. 2007; Gokoglu et al. 2004; Yu et al. 2020). Comparable findings were also observed in fried catfish, where mineral concentrations increased significantly following thermal processing (Marimuthu et al. 2011).

### 3.7 Textural Analysis During Frying of Hilsa Fish

The textural properties of Hilsa fish were significantly affected by frying time (*p* < 0.05), reflecting structural modifications induced by heat treatment and moisture loss (Table 7).

**TABLE 7:** Textural analysis of Hilsa fish.

Texture parameter	Frying time				
	0 min	3 min	6 min	9 min	12 min
Hardness (N)	12.50 ± 0.61^e^	18.72 ± 0.74^d^	24.31 ± 0.82^c^	30.18 ± 0.91^b^	36.52 ± 1.03^a^
Chewiness (N·mm)	9.81 ± 0.52^e^	14.23 ± 0.63^d^	18.49 ± 0.72^c^	23.07 ± 0.83^b^	28.69 ± 0.91^a^
Springiness (mm)	4.21 ± 0.12^a^	3.91 ± 0.13^b^	3.63 ± 0.14^c^	3.21 ± 0.12^d^	2.91 ± 0.11^e^
Cohesiveness	0.82 ± 0.02^a^	0.78 ± 0.02^b^	0.74 ± 0.02^c^	0.69 ± 0.02^d^	0.65 ± 0.02^e^
Gumminess (N)	10.33 ± 0.54^e^	14.57 ± 0.64^d^	17.91 ± 0.72^c^	20.88 ± 0.81^b^	23.73 ± 0.92^a^

*Note:* The mean ± standard deviation of three replicates is used to express the values. A substantial difference is shown by values inside each row that don't have the same superscript (*p* < 0.05).

Hardness increased significantly (*p* < 0.05) with frying time, indicating progressive firming of the muscle tissue. This change is associated with moisture loss, protein denaturation, and the formation of a crust layer on the surface. The development of a crispy texture in fried foods is primarily attributed to physicochemical transformations such as protein coagulation and structural shrinkage at the cellular level (Rahman et al. 2016).

Similarly, chewiness increased significantly (*p* < 0.05), corresponding to the enhanced firmness and reduced moisture content of the fish. The reduction in water content increases mechanical strength and results in a denser and more compact structure, which contributes to the characteristic texture of fried products (Singh et al. 2015).

In contrast, springiness decreased significantly (*p* < 0.05), indicating a reduced ability of the tissue to recover its original shape after deformation. Cohesiveness also showed a significant decline (*p* < 0.05), suggesting weakening of the internal structure due to protein denaturation and breakdown of connective tissues. These changes are consistent with the effects of high‐temperature frying, where protein cross‐linking, collagen shrinkage, and loss of water‐binding capacity reduce elasticity and structural integrity (Ishiwatari et al. 2013).

Gumminess increased significantly (*p* < 0.05), reflecting the combined effects of increased hardness and partial retention of cohesiveness. This indicates that although the structure becomes firmer, some degree of internal binding remains. Similar trends in textural properties have been reported in thermally processed fish and surimi products (Yu et al. 2020).

### 3.8 Sensory Evaluation of Fried Hilsa Fish

**FIGURE 1:**
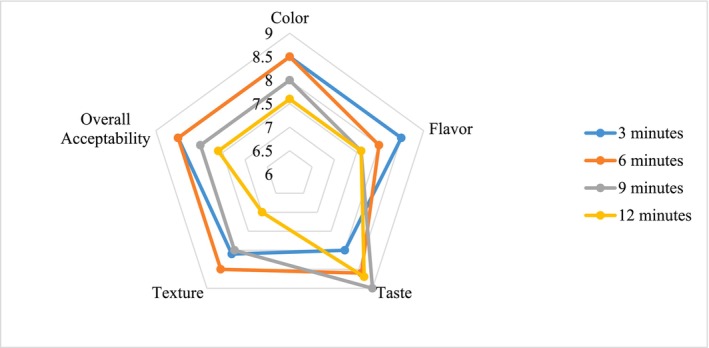
Sensory evaluation of fried Hilsa fish.

The sensory evaluation of fried Hilsa fish samples, assessed using a 9‐point hedonic scale, showed significant differences (*p* < 0.05) among frying times for key attributes including color, flavor, taste, texture, and overall acceptability (Figure 1). These variations reflect the combined effects of thermal processing on physicochemical and sensory properties.

Moderate frying durations (3–6 min) resulted in the highest scores for color and overall acceptability, indicating that these conditions produced a desirable golden‐brown appearance and balanced sensory quality. This outcome is likely associated with optimal Maillard browning and controlled moisture loss, which enhance visual appeal without excessive darkening.

Flavor, a critical determinant of consumer preference, was highest at shorter frying times, suggesting that limited heat exposure helps preserve the natural aroma and characteristic taste of Hilsa. In contrast, prolonged frying led to a decline in flavor scores, which may be attributed to lipid oxidation and thermal degradation of flavor compounds, resulting in the development of off‐flavors.

Taste and texture exhibited a slightly different trend, with higher scores observed at longer frying durations. This improvement is likely due to increased crispiness and firmness resulting from moisture loss and structural changes in the fish tissue. However, excessive frying may reduce juiciness and negatively impact overall sensory balance, as reflected in the decline of other attributes.

The observed decline in sensory quality with extended frying time is consistent with previous studies, which reported that prolonged frying deteriorates color, flavor, odor, and overall acceptability due to progressive thermal damage (Tadesse et al. 2020; Pawar et al. 2013). Lipid and protein oxidation during frying can generate undesirable compounds such as free fatty acids, peroxides, and free radicals, negatively affecting flavor and aroma (Wang et al. 2023). Additionally, excessive non‐enzymatic browning and oil degradation contribute to reduced visual appeal and sensory acceptance (Tadesse et al. 2020). Texture remains a key factor influencing consumer satisfaction, as it directly affects mastication and overall eating experience (Del Carmen Flores‐Álvarez et al. 2012).

The findings of this study have important dietary and nutritional implications. Hilsa fish is valued as a source of high‐quality protein, essential amino acids, minerals, and health‐promoting n‐3 polyunsaturated fatty acids. However, the present results show that prolonged frying can reduce some of these nutritional advantages by decreasing beneficial fatty acids such as eicosapentaenoic acid (EPA), altering amino acid composition, and modifying fat quality indices through increased oxidation. Although the apparent mineral content increased during frying, this was largely associated with moisture loss and concentration effects rather than an actual gain in nutrients. From a dietary perspective, these results suggest that frying time should be carefully controlled to balance sensory acceptability with nutritional preservation. Based on both compositional and sensory findings, moderate frying durations of 3–6 min appear to provide a more favorable compromise between desirable eating quality and retention of nutritional value. In addition, the choice of frying oil is important, since oils with greater oxidative stability may better preserve lipid quality during frying.

### 3.9 Limitations of the Study

Despite providing valuable insights into the effects of frying duration on the physicochemical and nutritional properties of Hilsa fish, this study has several limitations. The sensory evaluation was conducted using a panel composed primarily of young participants, which may not fully represent the preferences of the broader population. Sensory perception and food preference can vary among different age groups, cultural backgrounds, and dietary habits. Therefore, the results of the sensory analysis should be interpreted with caution when generalizing to the entire consumer population. Future studies should include a more diverse panel consisting of participants from different age groups and demographic backgrounds to obtain more representative sensory evaluation results.

## 4 Conclusion

This study demonstrates that frying time is a critical factor influencing the physicochemical characteristics, nutritional composition, and sensory quality of Hilsa ( *T. ilisha* ). Frying induced significant moisture loss and increased fat content due to oil absorption, whereas dry‐basis protein content declined, indicating thermal degradation. Prolonged frying markedly reduced essential amino acids, particularly lysine and histidine, and caused substantial losses of nutritionally important n‐3 polyunsaturated fatty acids including eicosapentaenoic acid (EPA). Concurrently, increases in saturated and trans fatty acids, along with elevated peroxide and acid values, confirmed progressive lipid oxidation and deterioration of fat quality. Textural and color changes further reflected heat‐induced structural modifications, with increased hardness and browning associated with moisture loss, protein denaturation, and Maillard reactions. Although mineral contents increased, this was primarily attributed to concentration effects rather than true nutritional enhancement. Sensory evaluation revealed that moderate frying durations (3–6 min) provided optimal balance between desirable flavor, texture, and overall acceptability, whereas extended frying negatively affected sensory attributes due to excessive oxidation and quality degradation.

## Author Contributions

**Arafat Hossain:** writing – original draft, investigation, formal analysis. **Md. Suman Rana:** conceptualization, supervision, formal analysis, writing – review and editing. **Rafiqul Islam:** investigation, formal analysis. **Md. Pallob Hossain:** writing – review and editing, formal analysis, writing – original draft. **Mrityunjoy Biswas:** writing – review and editing, validation. **Shafi Ahmed:** writing – review and editing, validation. **Sharmin Akther:** writing – review and editing, formal analysis. **Bhuiyan Mohammad Khaled:** writing – review and editing, validation. **Md. Momin Khan:** writing – review and editing, formal analysis.

## Funding

The authors have nothing to report.

## Ethics Statement

This study did not involve human participants or live animals except in sensory evaluation. Sensory evaluation involving human participants was conducted with voluntary informed consent, and all panelists were briefed on the research objectives and procedures. At present, there are no formal national ethical standards specifically governing sensory evaluation research in Bangladesh. However, the study protocol was reviewed and approved by the Committee of Department of Food Engineering, Jashore University of Science and Technology.

## Conflicts of Interest

The authors declare no conflicts of interest.

## Data Availability

The data that support the findings of this study are available from the corresponding author upon reasonable request.
